# Concurrent Validity of Three Photogrammetric Methods for Assessing Knee Alignment in Sagittal Plane

**DOI:** 10.3390/mps8020041

**Published:** 2025-04-14

**Authors:** Bruna Nichele da Rosa, Paula Andryelly Gomes Giendruczak, Marina Ziegler Frantz, Matias Noll, Cláudia Tarragô Candotti

**Affiliations:** 1 Physical Education Department, Federal University of Rio Grande do Sul, Porto Alegre 90010-150, Brazil; paula.giendruczak@ufrgs.br (P.A.G.G.); marina.frant@ufrgs.br (M.Z.F.); claudia.candotti@ufrgs.br (C.T.C.); 2 Nutrition and Health Department, Federal University of Goiás, Goiania 74690-900, Brazil; 3 Public Health Department, Federal Institute Goiano, Ceres 76300-000, Brazil

**Keywords:** knee, posture, photogrammetry, reproducibility of tests

## Abstract

*Background*: Evidence supporting the validity of photogrammetry for assessing body segment alignment remains limited, with most studies focusing on spinal evaluation. Thus, there is a lack of robust research examining its use for other body segments such as the lower limbs. *Objective*: This study aimed to evaluate the concurrent validity of three photogrammetric methods for measuring knee alignment in the sagittal plane with and without corrections for potential rotational deviations in the participant’s thigh and leg. *Methods*: A total of 21 adults underwent sequential evaluations involving panoramic radiography of the lower limbs and photogrammetry at a private radiology clinic. Photogrammetric analysis involved identifying the following anatomical landmarks: the greater trochanter of the femur (GTF), the lateral condyle of the femur (LCF), the head of the fibula (HF), and lateral malleolus (LM). Three photogrammetric methods were employed: (1) the condylar angle (CA) defined by the GTF, LCF, and LM points; (2) the fibula head angle (FHA) defined by the GTF, HF, and LM points; and (3) the four-point angle (4PA) incorporating the GTF, LCF, HF, and LM. Concurrent validity was assessed using correlation analysis, agreement with radiographic measurements, and the root mean square error (RMSE). Each photogrammetric method was tested using raw (CA, FHA, and 4PA) and corrected (CAcorr, FHAcorr, and 4PAcorr) values, accounting for thigh and/or leg rotational deviations. *Results*: Correcting for thigh and leg rotations significantly improved the validity metrics for all methods. The best performance was observed with the corrected condylar angle (CAcorr: r = 0.746; adjusted r^2^ = 0.533; RMSE = 2.9°) and the corrected four-point angle (4PAcorr: r = 0.733; adjusted r^2^ = 0.513; RMSE = 3.0°); however, the measurements presented proportional errors, possible due the method of assessment of rotations. *Conclusions*: The findings validate the evaluated photogrammetric methods for assessing sagittal knee alignment. Accounting for thigh and leg rotational deviations is critical for achieving accurate measurements, raising the need of accurate tools for measuring rotational changes in the lower limbs to avoid errors.

## 1. Introduction

Photogrammetry has become increasingly prominent in postural assessments because of its low cost and accessibility compared with the gold-standard method [1,2]. However, the validity of this alternative approach has yet to be firmly established [1]. Although photo-based quantitative posture analysis tools are available, the measurements that they provide have not been adequately validated [3,4].

The limited available studies have primarily focused on spinal evaluations [5,6,7,8,9], with a few studies addressing other body segments, such as sagittal knee alignment [10,11]. Thus, achieving a comprehensive postural assessment using photogrammetry based on a fully validated protocol is challenging, because the validity of assessments for all body segments has not been established. Similarly, it is not feasible to independently assess body segments other than the spine due to the lack of validated methodologies.

A systematic review and meta-analysis (Section 1) identified only two studies that examined the validity of photogrammetry for assessing sagittal knee alignment [10,11]. However, neither study used the gold-standard method as a reference. Instead, alternative tools such as goniometry and kinematics were employed to test the concurrent validity of photogrammetry. In validity studies, the choice of reference instrument is critical [12]. Among the dimensions of measurement validity, concurrent validity specifically refers to the “degree to which an instrument adequately reflects the gold standard” [13]. The gold standard for sagittal knee alignment assessments is the determination of the mechanical axis of the knee, which is based on the mechanical axes of the femur and tibia. This can be accurately performed using panoramic radiographs of the lower limbs [14,15]. To the best of our knowledge, no study has tested the validity of photogrammetry for assessing sagittal knee alignment using the gold-standard method as a reference. Another important consideration when using two-dimensional tools such as photogrammetry to assess knee alignment in the sagittal plane is the potential for rotational changes in the thigh and leg segments [16]. These segments are critical for accurately measuring the sagittal angle of the knee, and their rotation can alter the positions of anatomical reference markers. Such positional changes can result in inaccurate evaluations of knee alignment in the sagittal plane, as discussed in Section 2. This study was designed considering the limited availability of methods validated against a gold-standard reference and the potential for measurement errors in existing methods due to their failure to account for transverse-plane rotations when assessing sagittal knee alignment. The objective of the present study was to evaluate the concurrent validity of three photogrammetric methods for measuring sagittal knee alignment with and without corrections for possible thigh and leg rotations.

## 2. Methods

### 2.1. Sample

This validation study adhered to the Standards for Reporting Diagnostic Accuracy (STARD) guidelines [12]. A non-probabilistic consecutive sample of adults aged 18–60 years was employed. Participants were excluded if they had knee and/or hip prostheses, were undergoing treatment with orthopedic insoles during the study period, or had a lower limb length discrepancy of ≥2 cm. This last criterion was determined because the discrepancy could cause (a) difficulty in remaining in the orthostatic position without the use of orthopedic insoles and (b) lateral tilt of the pelvis, which could have some effect on hip rotation.

The sample size was calculated using the G*Power 3.1.9.2 software by applying the Z family of tests (Pearson’s correlation test). The calculation assumed a null hypothesis correlation of zero and an expected very strong correlation of 0.75 between the photogrammetric and radiological evaluations [17], with a significance level of 5% and a statistical power of 80%. This resulted in the required sample size of 18 individuals. All participants signed an informed consent form before participating, and the study was approved by the Research Ethics Committee of the affiliated university (approval no. 4,084,654).

### 2.2. Data Collection Procedure

The data were collected at a radiology clinic in Porto Alegre, Rio Grande do Sul, Brazil. Before the photogrammetry and radiographic evaluations, the participants underwent anamnesis to confirm the eligibility criteria and were assessed for anthropometric characteristics (body mass and height) and lower limb length to identify potential discrepancies.

**Figure 1:**
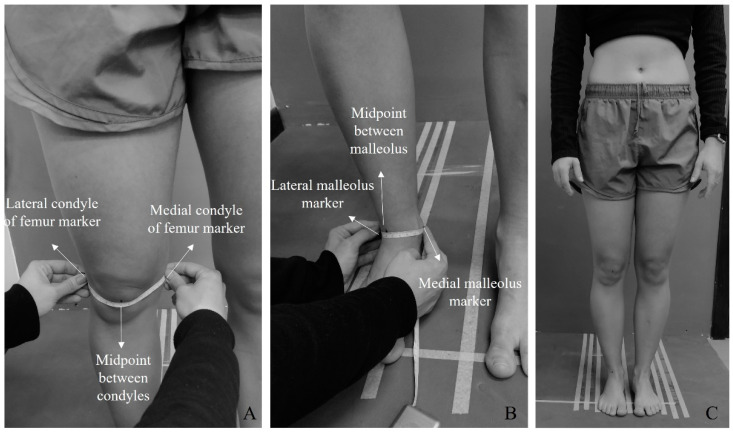
Marking of reference points to assess the presence of rotations in the thigh (**A**) and leg (**B**). The participant’s position during evaluation (**C**).

After being confirmed to satisfy the eligibility criteria, the participants were assessed for the presence of rotational changes in the thigh and leg. Because no validated or reproducible clinical method has been identified for quantifying thigh and leg rotations in a standing, weight-bearing position, the assessment was performed both visually and qualitatively. It involved marking the central points on the femoral condyles and malleoli with a dermatographic pencil (Figure 1A,B). The participants were then instructed to stand with their lower limbs together, respecting their natural anatomical alignments (Figure 1C). According to the positions of the marked central points, the thigh and leg were classified separately into one of three categories: internal rotation, external rotation, or neutral position. Based on the central point of the femoral condyles, we classified the femur into internal rotation if the central point was displaced medially, external rotation if the central point was displaced laterally, and neutral if the central point was faced anteriorly. The same criteria were used to classify the leg’s rotation, which was based on the central point of malleoli. Following this assessment, the participants underwent photogrammetry and panoramic radiography of the lower limbs. The order of these evaluations was randomized to reduce potential bias.

**Figure 2:**
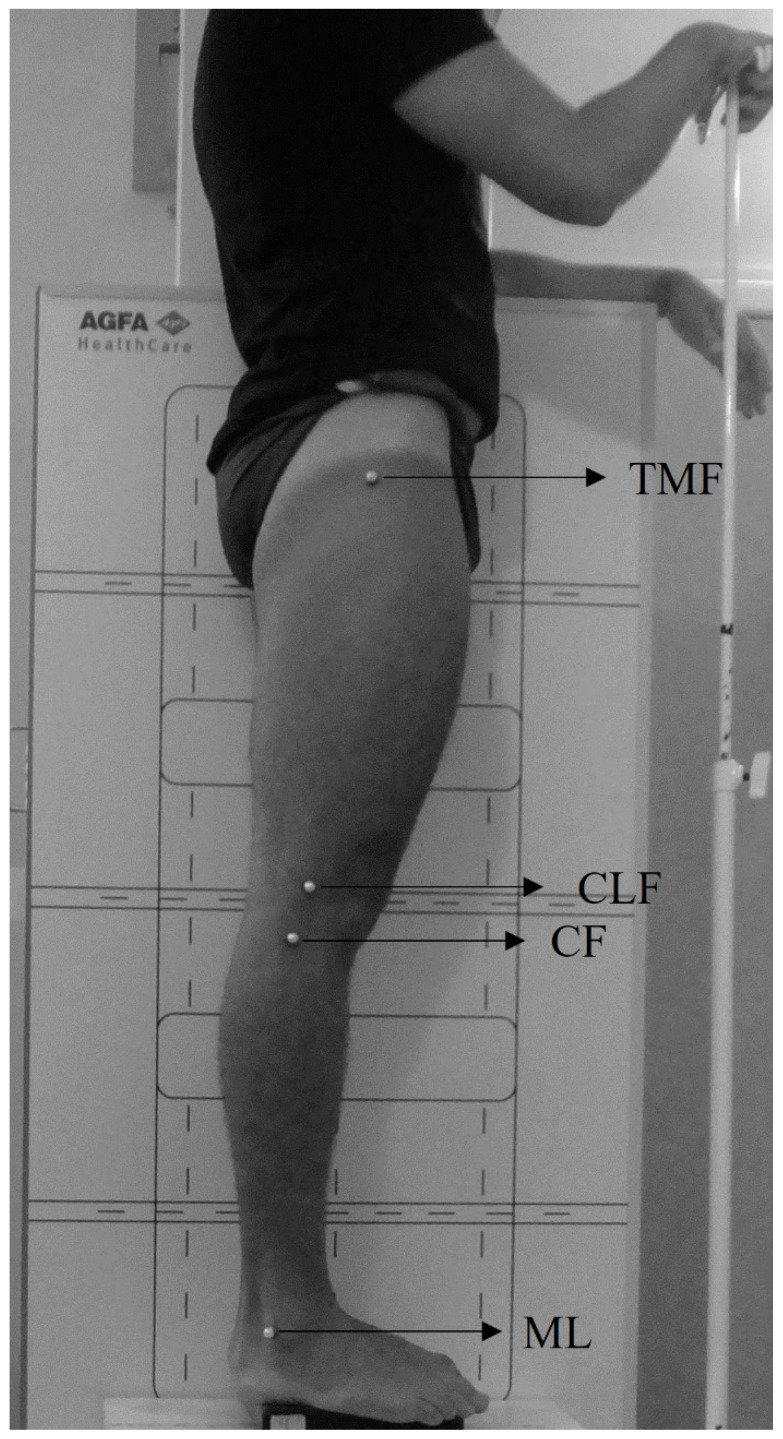
Anatomical landmarks used in the photogrammetric evaluation of the knee: GTF, LCF, HF, and LM. Positioning of participants for the photogrammetry and radiographic evaluations is also depicted.

Photogrammetric evaluation involves palpating and marking anatomical landmarks using adhesive spherical markers. Markers were placed on the greater trochanter of the femur (GTF), the lateral condyle of the femur (LCF), the head of the fibula (HF), and lateral malleolus (LM), as shown in Figure 2. The participants were then positioned with their right side facing the camera and lower limbs together [18], aligned according to their natural anatomy, and their right elbow was passively flexed (Figure 2). Photogrammetric evaluation was conducted at the same location and participant position as the radiographic evaluation (Figure 2).

The photogrammetric assessment was performed by a physical therapist with six years of experience in postural analysis and photogrammetry. For the radiographic evaluation, a lateral panoramic radiograph of the lower limbs was obtained by a certified radiology technician from the clinical team. The reference markers used in the photogrammetric evaluation were adhered to the participants’ skin during the radiographic procedure. To ensure safety, the participants wore protective equipment, including a thyroid shield and a pelvic protector, during the radiography session.

### 2.3. Data Analysis Procedure

**Figure 3:**
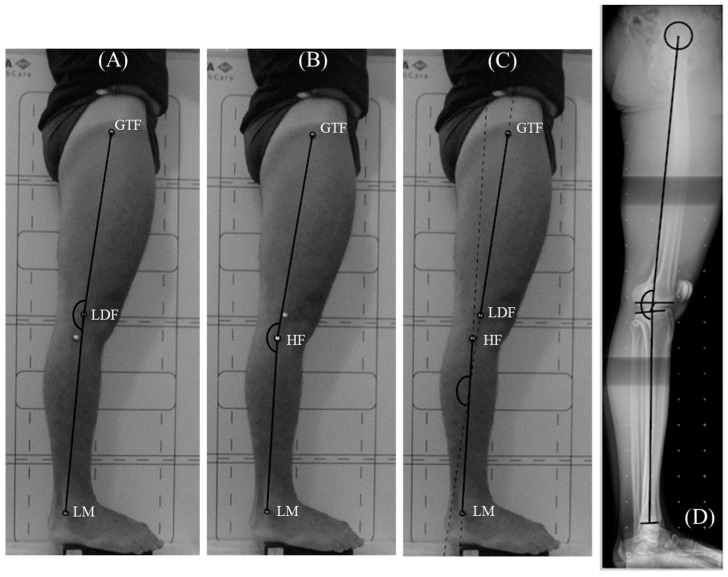
Sagittal knee angles calculated via photogrammetry: (**A**) condylar angle (CA); (**B**) fibula head angle (FHA); (**C**) four-point angle (4PA); and X-rays (**D**).

The images from both the photogrammetric and radiographic evaluations were analyzed using *SketchUp Pro* 2021 (Trimble Navigation, Sunnyvale, CA, USA)—a software program designed for three-dimensional modeling. In the photogrammetric evaluation, three sagittal knee angles were measured to identify the calculation method that achieved the highest validity indices: (a) the condylar angle (CA) formed by straight lines connecting the GTF, LCF, and LM points; (b) the fibula head angle (FHA) formed by straight lines connecting the GTF, HF, and LM points; and (c) the four-point angle (4PA) formed by straight lines connecting the GTF, LCF, HF, and LM points, as shown in Figure 3.

The sagittal knee angle on the radiographs was determined using the mechanical axes of the femur and tibia. The mechanical axis of the femur is defined as the line connecting the center of the femoral head to the center of the intercondylar fossa [14]. The mechanical axis of the tibia extends from the center of the intercondylar fossa to the center of the distal tibia [14]. The knee angle on radiography was defined as the posterior angle formed by the intersection of the femoral and tibial mechanical axes (Figure 3D).

To ensure blinding and reduce potential bias, the photogrammetric and radiographic images were analyzed by separate evaluators. Both evaluators were trained in advance to effectively use the image analysis software.

The validity of the raw photogrammetric values for each of the three sagittal angles (CA, FHA, and 4PA) was assessed, along with the values corrected for thigh and/or leg rotations observed during the qualitative evaluation (CAcorr, FHAcorr, and 4PAcorr). The correction values were derived from a previous study that employed a biomechanical model simulating the right lower limb of an adult and compared the knee sagittal angle assessment with different degrees of internal and external rotation of the thigh and leg. This study demonstrated that thigh and leg rotations significantly affect the measurement of sagittal knee alignment. A previous study tested the same three methods (CA, FHA, and 4PA) for calculating sagittal knee angles, providing correction values for each method.

To determine the correction values for the observed alterations in the body segments, the averages of the deviations in sagittal knee angles were calculated across ten biomechanical simulations, with five biomechanical simulations of external rotations (10–50°) and five simulations of internal rotations (10–50°) of the thigh and leg. These simulations used a biomechanical model built using two wooden rafters, each having a length of 40 cm and a width of 8 × 8 cm^2^. The rafters were positioned one above another. The upper rafter simulated the thigh, and the lower rafter simulated the leg. The sagittal angle deviations were calculated relative to the neutral position (without thigh or leg rotation). The correction values derived from the simulations are presented in Table 1. If a participant’s thigh or leg segment was classified as neutral (i.e., without rotation), the correction value for that segment was 0. For participants with internal or external rotation, corrections were applied using the same equation, with specific values based on the degree of rotation, as shown in Table 1. The general correction equation for all adjusted sagittal knee angles was as follows:

Angle_corrected_ = Angle_raw_ + correction_thigh_ (degrees) + correction_leg_ (degrees).

**Figure 4:**
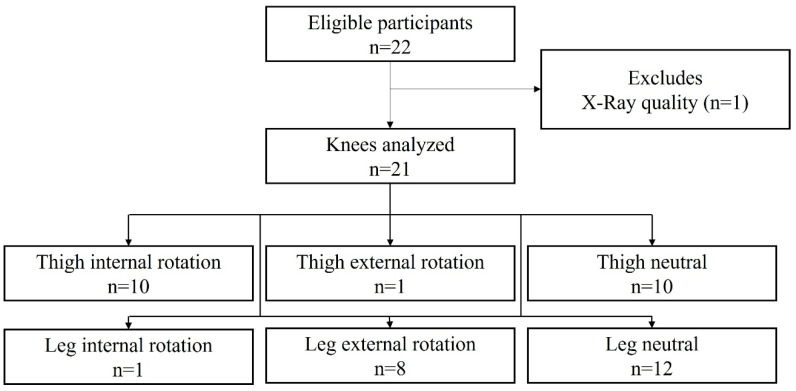
Flowchart illustrating participant characteristics regarding the presence or absence of thigh and/or leg rotations.

From this perspective, the participants with and without thigh and/or leg rotations were evaluated. Figure 4 presents a flowchart of the participants included in this study, categorized according to the transverse alignment characteristics of their lower limbs.

### 2.4. Statistical Analysis

Statistical analyses were conducted using the SPSS software, version 26 (IBM, Armonk, NY, USA). Descriptive statistics were used to characterize the participants. Data normality was assessed using the Shapiro–Wilk test. Pearson’s product–moment correlation test was used to evaluate the correlation between the angles obtained from photogrammetry (raw and corrected) and radiography. The correlation strength was classified as follows: “very low” (<0.1), “low” (0.1–0.3), “moderate” (0.3–0.5), “high” (0.5–0.7), “very high” (0.7–0.9), and “almost perfect” (>0.9). The Bland–Altman plot was used to assess the agreement between the photogrammetry (raw and corrected) and radiography values, presenting the proportional errors based on the ordinary least products (OLP) regression analysis and the fixed errors based on the mean difference. Additionally, Student′s *t*-test was performed to evaluate the differences between photogrammetry (raw and corrected) and radiography measurements.

Simple linear regression analysis was used to estimate the sagittal knee angle, which is considered the gold standard for photogrammetry measurements. For the method to be considered valid, it needed to meet the following criteria: a correlation coefficient of ≥0.7 and a coefficient of determination (R^2^) of ≥0.5. The root mean square error (RMSE) was calculated to assess the accuracy of photogrammetry. A significance level of 0.05 was adopted for all statistical analyses.

## 3. Results

A total of 22 adults participated in the study. One participant was excluded because of poor radiographic image quality, which hindered the analysis. The participants had a mean age of 28.4 ± 1.9 years, a mean body mass of 70.7 ± 2.3 kg, and a mean height of 171.3 ± 1.7 cm.

Table 2 presents the mean values of the sagittal knee angles obtained from radiography and photogrammetry (both raw and corrected for rotation), as well as the mean differences, correlation coefficients, and RMSEs for each photogrammetric method compared with radiography. The results indicated that except for the FHAcorr method, all the photogrammetric methods produced angular measurements comparable to those obtained from radiography. Additionally, these methods exhibited strong to very strong correlations with radiography, with RMSEs of <4°. Among them, the CAcorr and 4PAcorr methods exhibited very strong correlations with radiography and had the smallest RMSE values.

**Figure 5:**
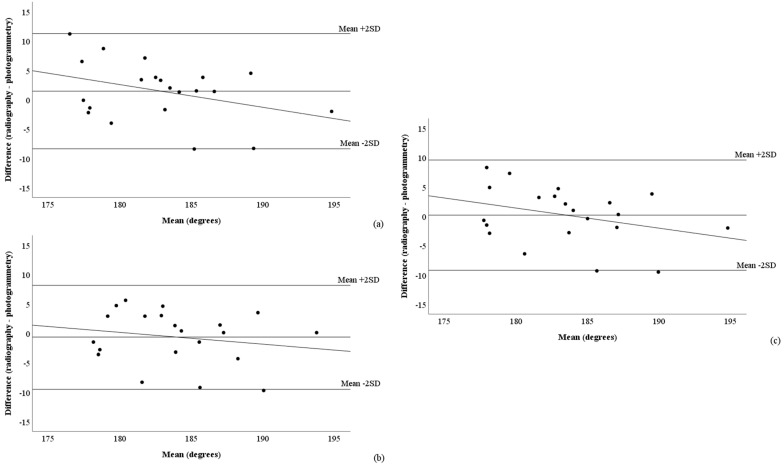
Bland–Altman graphical analysis showing the agreement between radiography and the three raw photogrammetric methods: (**a**) condylar angle, (**b**) fibula head angle, and (**c**) four-point angle. SD: standard deviation. Proportional errors: slope differs from 0 at *p* ≤ 0.05; fixed errors: mean difference differs from 0 at *p* ≤ 0.05.

**Figure 6:**
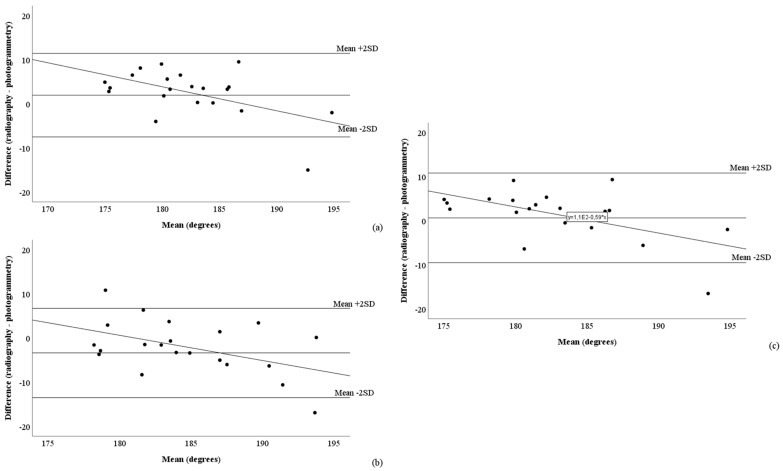
Bland–Altman graphical analysis showing the agreement between radiography and the three corrected photogrammetric methods: (**a**) corrected condylar angle, (**b**) corrected fibula head angle, and (**c**) corrected four-point angle. SD: standard deviation. Proportional errors: slope differs from 0 at *p* ≤ 0.05; fixed errors: mean difference differs from 0 at *p* ≤ 0.05.

Figure 5 and Figure 6, as well as Table 3, present the Bland–Altman analyses assessing the agreement between radiography and the raw (Figure 5) and corrected (Figure 6) photogrammetric methods. Among the photogrammetric methods that exhibited the best correlations with radiography (CAcorr and 4PAcorr), the mean differences relative to radiography were 0°, with limits of agreement (±2 standard deviations) around 10° (CAcorr: –7.5° to 11.4°; 4PAcorr: –9.5° to 10.8°). Only one measurement was outside the limits of agreement (Figure 6a,c). However, all methods using the correction equation presented proportional errors (Table 3).

Table 4 presents the results of the simple linear regression analysis, including the equations for estimating radiographic angles using photogrammetric methods and their coefficients of determination (adjusted r^2^). The CAcorr and 4PAcorr methods, corrected for thigh and/or leg rotations, achieved the highest coefficients of determination (adjusted r^2^ > 0.5), indicating strong predictive accuracy.

## 4. Discussion

This study aimed to evaluate the concurrent validity of three photogrammetric methods for assessing sagittal knee alignment with and without corrections for rotations in the thigh and/or leg segments of participants. At the completion of this study, no method available in the literature for measuring the sagittal angle of the knee has conclusively demonstrated both validity and reproducibility. Among the methods tested for validity, none employed the gold standard as a reference [10,11], which is essential for studies assessing concurrent validity [12].

All the methods tested exhibited significant and strong correlations with radiography, producing values close to those obtained using the gold standard, except for the FHAcorr method. The RMSE values for all the methods were <4°. Given that the physiological alignment of the knee in full extension is approximately 180° (Table 2) [19], error values below 4° were considered small. The FHAcorr method demonstrated a weak correlation to the gold standard, possibly due to the anatomical points involved in the measurement. It is important to note that the knee joint is formed by the femoral and tibial bones, and the fibula is not part of the knee [20,21]. Consequently, the measurements obtained from this anatomical point are not aligned with the x-ray measurements, which utilize the mechanical axis of the femur and tibia [14]. The mechanical axis of the femur is delineated as the line connecting the center of the femoral head to the center of the intercondylar fossa [14]. The mechanical axis of the tibia extends from the center of the intercondylar fossa to the center of the distal tibia [14]. Consequently, as indicated by the findings of our study, it is not recommended that the photogrammetry tools utilize this measurement as a sagittal knee angle given its lack of correlation with the gold standard.

Among the methods tested, two exhibited very strong correlations with radiography: the CAcorr and 4PAcorr methods. The Bland–Altman graphical analysis revealed that these methods agreed well with radiography, as the data were well dispersed within the limits of agreement, with only one measurement falling outside these limits. In addition, the mean difference between the methods and radiography was approximately zero [22,23]. However, these two methods presented proportional errors, despite presenting lower RMSE values (Table 2) and higher coefficients of determination (Table 4). The proportional errors can be associated with the indirect and qualitative measurement of rotational changes, since only methods using this correction presented the proportional error, a limitation of this study.

It is important to note that rotations in the thigh and leg significantly affect the measurement of the sagittal knee angle, either increasing or decreasing the angle, which can result in the inaccurate classification of the knee posture. Therefore, given that knee measurements are affected by transverse plane changes (rotations), caution is required when using two-dimensional instruments, particularly those that do not account for rotational variations.

To increase the accuracy of sagittal knee assessments, it is advisable to complement them with evaluations that identify and quantify potential rotations in the thigh and leg segments. Incorporating these values into the sagittal knee angle measurement process enhances the reliability of the assessment. Although computed tomography is the gold standard for evaluating femoral and tibial rotation [16,24], its high cost and limited accessibility pose significant barriers to its routine use. Furthermore, to the best of our knowledge, no clinical method that quantifies thigh and leg rotations in the same position used for photogrammetric evaluation exists, i.e., with the patient standing and bearing weight on the lower limbs.

While this approach offers a practical alternative in the absence of validated and reliable clinical methods for measuring lower limb rotation, it is not without limitations. One notable limitation of this study was the lack of quantitative measurements of thigh and/or leg rotation magnitudes. Qualitative evaluation is inherently subjective and relies heavily on the evaluator’s expertise [25], which may lead to incorrect correction values if the participants are evaluated inaccurately. Therefore, it is notable that new methods for clinically assessing and quantifying lower limb rotations are needed, since the qualitative method used in this study can be the source of proportional errors in the measurement.

Additionally, this study’s sample size was imbalanced regarding the classification of thigh and leg rotations, with only one participant presenting with external rotation of the thigh and one presenting with internal rotation of the leg (Figure 4). Thus, the findings of this study were constrained by the limited sample size and the uneven distribution of participants with different lower limb rotation characteristics. This restricts the ability to fully demonstrate the validity of the tested methods across the entire spectrum of lower limb rotational variations.

## 5. Conclusions

From the findings of this study, it can be concluded that among the three photogrammetric methods tested, two methods—the corrected condylar angle (CAcorr) and corrected four-point angle (4PAcorr)—were validated. These methods were corrected for the presence of thigh and/or leg rotations. Therefore, the presence of rotations in the thigh and leg should be assessed when using photogrammetry for sagittal knee evaluations. Incorporating the correction values identified in this study is essential for accurately calculating the sagittal knee angle. However, it is important to highlight the caution of using qualitative techniques for assessing the presence of rotation in lower limbs, as it can produce proportional errors in the measurements, as shown in this study.

## Figures and Tables

**Table 1:** Values used for the correction of sagittal knee angles in photogrammetry due to rotations in the thigh and/or leg.

Measurement Method	Correction by Thigh Rotation		Correction by Leg Rotation	
	Internal Rotation	External Rotation	Internal Rotation	External Rotation
Condylar angle (CA)	3.1°	–5.2°	3.6°	–5.0°
Fibula head angle (FHA)	6.3°	–7.8°	0.9°	0.1°
Four-point angle (4PA)	3.7°	–5.7°	3.3°	–5.5°

**Table 2:** Mean and SD (n = 21), Pearson’s correlation coefficient (r), Student’s *t*-test (*t*), and root mean square error (RMSE) of photogrammetry in relation to radiography.

Measurement Method	Average ± SD	r	Average Difference (95% CI) (Radiography–Photogrammetry)	*t*	RMSE
Radiography	183.6 ± 1	-	-	-	-
CA	182.2 ± 1.3	0.586 (*p* = 0.005)	1.4 (–0.8 to 3.7)	1.326 (*p* = 0.200)	3.5
FHA	184.3 ± 1.1	0.572 (*p* = 0.007)	–0.6 (–2.7 to 1.4)	–0.656 (*p* = 0.519)	3.6
4PA	183.4 ± 1.3	0.6 (*p* = 0.004)	0.2 (–2 to 2.4)	0.178 (*p* = 0.861)	3.5
CA_corr_	181.7 ± 1.5	0.746 (*p* < 0.001)	–1.9 (–4.1 to 0.2)	–1.853 (*p* = 0.079)	2.9
FHA_corr_	187 ± 1.4	0.595 (*p* = 0.004)	3.4 (1 to 5.7)	**3.019 (*p* = 0.007)**	3.5
4PA_corr_	183 ± 1.6	0.733 (*p* < 0.001)	–0.6 (–3 to 1.7)	–0.561 (*p* = 0.581)	3

SD, standard deviation; 95% CI, 95% confidence interval; RMSE, root mean square error; CA, raw condylar angle; FHA, raw fibular head angle; 4PA: raw four-point angle; CAcorr, corrected condylar angle; FHAcorr, corrected fibular head angle; 4PAcorr: corrected four-point angle.

**Table 3:** Outcomes of analyses of differences by ordinary least squares regression for measured proportional errors.

Regression	r	a	b	*p*
(XR − CA)/(meanXRCA)	0.364	−0.39	72.21	0.105
(XR − FHA)/(meanXRFHA)	0.109	−0.20	36.27	0.409
(XR − 4PA)/(meanXR4PA)	0.331	−0.34	63.06	0.143
(XR − CA_corr_)/(meanXRCA_corr_)	0.534	−0.54	**101.61**	**0.013**
(XR − FHA_corr_)/(meanXRFHA_corr_)	0.471	−0.57	**102.69**	**0.031**
(XR − 4PA_corr_)/(meanXR4PA_corr_)	0.560	−0.59	**109.66**	**0.008**

XR, x-ray; CA, raw condylar angle; FHA, raw fibula head angle; 4PA, raw four-point angle; CAcorr, corrected condylar angle; FHAcorr, corrected fibula head angle; 4PAcorr, corrected four-point angle; r, product–moment correlation coefficient; a, b (slope), coefficients in ordinary least squares regression model E (x-ray – photogrammetry) = a + b(mean xrayphotogrammetry); proportional error if b differs significantly from 0 (*p* ≤ 0.05).

**Table 4:** Radiography prediction equations and coefficients of determination (adjusted r^2^) for each photogrammetric method tested.

Measurement Method	Equation for Estimating the Radiographic Angle	Adjusted r^2^
CA	Â_rad_ = 105.386 + 0.429(Â_photo_)°	0.309
FHA	Â_rad_ = 93.710 + 0.488(Â_photo_)°	0.291
4PA	Â_rad_ = 100.259 + 0.454(Â_photo_)°	0.326
CA_corr_	Â_rad_ = 99.024 + 0.466(Â_photo_)°	0.533
FHA_corr_	Â_rad_ = 105.713 + 0.417(Â_photo_)°	0.321
4PA_corr_	Â_rad_ = 103.628 + 0.437(Â_photo_)°	0.513

CA, raw condylar angle; FHA, raw fibula head angle; 4PA, raw four-point angle; CAcorr, corrected condylar angle; FHAcorr, corrected fibula head angle; 4PAcorr, corrected four-point angle; rad, radiographic angle; photo, photogrammetric angle.

## Data Availability

On requesting to the authors.

## References

[1] Do Rosário J.L.P. (2014). Photographic analysis of human posture: A literature review. J. Bodyw. Mov. Ther..

[2] Fortin C., Feldman D.E., Cheriet F., Labelle H. (2011). Clinical methods for quantifying body segment posture: A literature review. Disabil. Rehabil..

[3] Ferreira E.A.G., Duarte M., Maldonado E.P., Burke T.N., Marques A.P. (2010). Postural Assessment Software (PAS/SAPO): Validation and Reliabiliy. Clinics.

[4] Paušić J., Pedišić Ž., Dizdar D. (2010). Reliability of a Photographic Method for Assessing Standing Posture of Elementary School Students. J. Manip. Physiol. Ther..

[5] Döhnert M.B., Tomasi E. (2008). Validity of computed photogrammetry for detecting idiopathic scoliosis in adolescents. Rev. Bras. Fisioter..

[6] Furlanetto T.S., Candotti C.T., Sedrez J.A., Dutra V.H., Vieira A., Loss J.F. (2020). Concurrent Validity of Digital Image-Based Postural Assessment as a Method for Measuring Thoracic Kyphosis: A Cross-Sectional Study of Healthy Adults. J. Manip. Physiol. Ther..

[7] Furlanetto T.S., Candotti C.T., Comerlato T., Loss J.F. (2012). Validating a postural evaluation method developed using a Digital Image-based Postural Assessment (DIPA) software. Comput. Methods Programs Biomed..

[8] Navarro I.J., Candotti C.T., Amaral M.A.D., Dutra V.H., Gelain G.M., Loss J.F. (2020). Validation of the Measurement of the Angle of Trunk Rotation in Photogrammetry. J. Manip. Physiol. Ther..

[9] Ruivo R.M., Pezarat-Correia P., Carita A.I. (2015). Intrarater and Interrater Reliability of Photographic Measurement of Upper-Body Standing Posture of Adolescents. J. Manip. Physiol. Ther..

[10] Hopkins B.B., Vehrs P.R., Fellingham G.W., George J.D., Hager R., Ridge S.T. (2019). Validity and Reliability of Standing Posture Measurements Using a Mobile Application. J. Manip. Physiol. Ther..

[11] Sacco I.D.C.N., Alibert S., Queiroz B.W.C., Pripas D., Kieling I., Kimura A.A., Sellmer A.E., Malvestio R.A., Sera M.T. (2007). Confiabilidade da fotogrametria em relação a goniometria para avaliação postural de membros inferiores. Rev. Bras. Fisioter..

[12] Bossuyt P.M., Reitsma J.B., Bruns D.E., Gatsonis C.A., Glasziou P.P., Irwig L.M., Moher D., Rennie D., De Vet H.C., Lijmer J.G. (2003). The STARD Statement for Reporting Studies of Diagnostic Accuracy: Explanation and Elaboration. Croat. Med. J..

[13] Mokkink L.B., Terwee C.B., Patrick D.L., Alonso J., Stratford P.W., Knol D.L., Bouter L.M., de Vet H.C. (2010). The COSMIN study reached international consensus on taxonomy, terminology, and definitions of measurement properties for health-related patient-reported outcomes. J. Clin. Epidemiol..

[14] Cebulski-Delebarre A., Boutry N., Szymanski C., Maynou C., Lefebvre G., Amzallag-Bellenger E., Cotten A. (2016). Correlation between primary flat foot and lower extremity rotational misalignment in adults. Diagn. Interv. Imaging.

[15] da Rosa B.N., Camargo E.N., Candotti C.T. (2022). Radiographic Measures for the Assessment of Frontal and Sagittal Knee Alignments and the Associated Normality Values: A Meta-Analysis. J. Chiropr. Med..

[16] Tamari K., Tinley P., Briffa K., Breidahl W. (2005). Validity and reliability of existing and modified clinical methods of measuring femoral and tibiofibular torsion in healthy subjects: Use of different reference axes may improve reliability. Clin. Anat..

[17] Hopkins W.G. (2018). A New View of Statistics: A Scale of Magnitudes for Effect Statistics. http://www.sportsci.org/resource/stats/.

[18] Antoniolli A., Candotti C.T., Gelain G.M., Schmit E.F.D., Ducatti L.M.A., Melo M.d.O., Loss J.F. (2018). Influence of feet position on static postural assessment by means of photogrammetry: A comparative study. Eur. J. Physiother..

[19] Furlanetto T.S., Candotti C.T., Sedrez J.A., Noll M., Loss J.F. (2017). Evaluation of the precision and accuracy of the DIPA software postural assessment protocol. Eur. J. Physiother..

[20] Flandry F., Hommel G. (2011). Normal Anatomy and Biomechanics of the Knee. Sports Med. Arthrosc. Rev..

[21] Moore A.F.D. (2018). Anatomia Orientada para a Clínica.

[22] Bland J.M., Altman D.G. (2010). Statistical methods for assessing agreement between two methods of clinical measurement. Int. J. Nurs. Stud..

[23] Giavarina D. (2015). Understanding Bland Altman analysis. Biochem. Medica.

[24] Durandet A., Ricci P.L., Saveh A.H., Vanat Q., Wang B., Esat I., Chizari M. (2013). Radiographic analysis of lower limb axial alignments. Lect. Notes Eng. Comput. Sci..

[25] Normand M.C., Descarreaux M., Harrison D.D., Harrison D.E., Perron D.L., Ferrantelli J.R., Janik T.J. (2007). Three dimensional evaluation of posture in standing with the PosturePrint: An intra- and inter-examiner reliability study. Chiropr. Osteopat..

